# *Lactobacillus bulgaricus* improves antioxidant capacity of black garlic in the prevention of gestational diabetes mellitus: a randomized control trial

**DOI:** 10.1042/BSR20182254

**Published:** 2019-08-09

**Authors:** Lihui Si, Ruixin Lin, Yan Jia, Wenwen Jian, Qing Yu, Min Wang, Shuli Yang

**Affiliations:** 1 Department of Gynecology and Obstetrics, The Second Hospital of Jilin University, Changchun 130000, China; 2 Department of Genral Surgery, The Second Hospital of Jilin University, Changchun 130000, China

**Keywords:** antioxidant capacity, black garlic, gestational diabetes, Lactobacillus bulgaricus, oral glucose tolerance test

## Abstract

**Objectives:** *Lactobacillus bulgaricus* may improve antioxidant capacity of black garlic in the prevention of gestational diabetes mellitus (GDM).

**Methods:** Black garlic was prepared with or without *L. bulgaricus*. Volatile and polysaccharides were analyzed by using LC-MS, Fourier Transform infrared (FTIR) and ^13^C nuclear magnetic resonance (NMR). The study design was parallel randomized controlled trial and 226 GDM patients were randomly assigned into BG (black garlic and *L. bulgaricus*) and CG (black garlic) groups, and allocation ratio was 1:1. The treatment duration was 40 weeks. Fasting blood glucose (FBG) and 1- and 2-h blood glucose (1hBG and 2hBG) after oral glucose tolerance test (OGTT) were detected. Antioxidant function of black garlic was determined by measuring plasma malondialdehyde (MDA), superoxide dismutase (SOD), glutathione peroxidase (GSH-PX) and total antioxidant capacity (T-AOC) in GDM patients. The comparison between two groups was made using two independent samples *t* test.

**Results:** The intake of nutrients was similar between two groups (*P*>0.05). *L. bulgaricus* promoted the transformation of the glucopyranoside to glucofuranoside. *L. bulgaricus* increased the abilities of black garlic for scavenging hydroxyl radicals, 2,2′-azino-bis (3-ethylbenzenthiazoline-6-sulfonic) acid (ABTS) and DPPH free radicals. *L. bulgaricus* reduced the levels of FBG, 1hBG and 2hBG, and incidence of perinatal complications (*P*<0.01). Plasma MDA level in the BG group was lower than in the CG group, whereas the levels of SOD, GSH-PX and T-AOC in the BG group were higher than in the CG group (*P*<0.01).

**Conclusions:** *L. bulgaricus* improves antioxidant capacity of black garlic in the prevention of GDM.

## Introduction

Gestational diabetes mellitus (GDM) refers to abnormal glucose tolerance during pregnancy [1]. GDM is potentially harmful to both mother and offspring, including increased amniotic fluid [2], preeclampsia [3], premature birth [4], ketoacidosis [5] and the development of type 2 diabetes mellitus (T2DM) [6] for mother, and abnormalities in intrauterine development [7], neonatal malformations [8], macrosomia [9], neonatal respiratory distress syndrome [10] and neonatal hypoglycemia [11] for offspring. At present, the study mainly focuses on GDM treatment after the middle and late pregnancy. However, there is very little research on early pregnancy intervention of GDM.

The ‘Guidelines for the Diagnosis and Treatment of Gestational Diabetes’ show the risk factors for GDM, including overweight, GDM history or relatives with T2DM [12]. Pregnant women with a history of infertility and polycystic ovary syndrome (PCOS) are at risk for developing GDM [13]. Fasting blood glucose (FBG) ≥ 5.1 mmol/l is an independent risk factor for GDM. FBG is often measured during the first trimester of pregnancy, and FBG levels can be one of risk factors for screening high-risk GDM. Epidemiological data showed that the incidence of GDM was positively correlated with the increase in body-mass index (BMI) during pregnancy [14].

Dietary interventions [15], exercise interventions [16], weight management [17] and blood glucose monitoring [18] will be beneficial to control GDM risk or improve its outcome. Black garlic is a normal Chinese herb with a compelling profile of bioactive components and potential anti-T2DM activities [19,20]. Black garlic has anti-inflammatory [21] and antioxidant functions [22]. Black garlic has anti-lipogenic and lipolytic activities and a potential in anti-obesity therapy [23]. On the other hand, probiotics can control glycemic level and reduce inflammation in GDM patients [24], and have antioxidant capacity [25]. GDM patients have abnormal lipid profiles based on physiologic subtype [26]. Hyperglycemia during pregnancy and inflammation are the main reasons for developing GDM complications [27]. Thus, probiotics may have complementary function with black garlic in the prevention of GDM.

## Materials and methods

### Preparation of black garlic

*L. bulgaricus*, the main bacteria used for yogurt, was purchased from Institute of Microbiology of the CAS (Beijing, China), and cultured in MRS medium at 37°C for 3 days. The probiotics were washed with distilled water for three times and suspended in distilled water. Fresh garlic (*Allium sativum*) was purchased from Jinxiang Supermarket (Jinxiang, China). The fresh garlic of the control group was naturally fermented (60°C, 70–95% relative humidity) for 50 days, then it was soaked in distilled water (1:2, w/v), and the fermentation continued for 7 days at 42°C to obtain black garlic. In *L. bulgaricus* group, black garlic was soaked in the solution with *L. bulgaricus* (10^8^ CFU/ml in distilled water, 1:2, w/v) for further fermentation at 42°C for 7 days. At the end of fermentation, garlic was washed out with sterile distilled water and dried in a 50°C incubator.

### Measurement of black garlic polysaccharides

Fresh garlic and deionized water were mixed at 1:1 (w/v), ground uniformly, ultrasonically disrupted (ultrasonic power 300 W, ultrasonic time 15 min), and then placed in a 90°C water bath for 2 h, and centrifuged at 10000×***g*** for 10 min. The supernatant was taken and the extraction was repeated three times. Garlic lipid was measured by an AdipoRed assay kit (Cambrex Bio Science, Walkersville, MD, U.S.A.). Total protein was measured by using a BCA assay kit (Pierce Biotechnology Inc, Rockford, IL, U.S.A.). Three-fold volume of ethanol was added to supernatants. The mixture was placed on ice for 12 h, centrifuged (10000×***g*** for 10 min), and the precipitate was dried at 55°C to obtain garlic polysaccharide.

The total sugar content was determined by the phenol-sulfuric acid method [28], and the reducing sugar content was determined by dinitrosalicylic acid (DNS) method [29]. Polysaccharide content was the difference between the total sugar content and the reducing sugar content. Briefly, different concentrations of sugar (0, 0.0125, 0.025, 0.0375, 0.05, 0.0625, 0.075, 0.0875, 0.1 mg/ml) were prepared in the test tubes, and then 1 ml phenol (6%) and 5 ml sulfuric acid were added, shaken thoroughly and allowed to stand for 20 min. The absorbing values were measured at 490 nm with a spectrophotometer, and a standard curve was drawn and the regression equation made. Two milliliters of sample was tested and 1 ml phenol was added to the test tube with triplicates. A total of 150 μl DNS was added to 150 μl reducing sugar in a 1.5-ml Eppendorf tube and reacted for 1 min at 37°C. Reducing sugar was measured at 420 nm as the release of reducing sugars from polysaccharide with one unit was defined as the release of 1 μmol sugar/min.

The structure of black garlic polysaccharide (BGP) was analyzed using ultraviolet (UV), Fourier Transform infrared (FTIR) and nuclear magnetic resonance (NMR). Two milligram BGP was dissolved in 20 mM phosphate buffer pH 7.4 and scanned via a UV-Vis spectrophotometer (PerkinElmer, Boston, MA, U.S.A.) from 200 to 400 nm. FTIR spectrum of BGP was measured from 500 to 4000 cm^−1^ in a spectrometer (Nicolet Instrument Corp., Madison, WI, U.S.A.); 0.1% BGP was prepared and KBr pallet was used to detect FTIR spectrum.

Cross-Polarization Magic Angle Spinning ^13^C NMR (CP/MAS ^13^C-NMR) was used to detect the structure of polysaccharide via a 320-MS spectrometer (Bruker Inc., Billerica, MA, U.S.A.) at a frequency of 75.5 MHz at 7.04 T static magnetic field. Carbon-13 spin lattice relaxation time was 5 s. BGP was positioned in a rotor and spun was set as 9 kHz.

### Measurement of BGP antioxidant capacity

The reducing ability of polysaccharides was measured by the Prussian blue method [30]. Garlic polysaccharide was diluted in a gradient method. A total of 1 ml sample, 2.5 ml phosphate buffer (0.2 mol/l, pH 6.6) and 1 ml of potassium ferricyanide solution (10 g/l) was uniformly mixed in a test tube. After being placed in a 50°C water bath for 20 min, 2 ml of trichloroacetic acid (100 g/l) and 1.2 ml of ferric chloride solution (1 g/l) were added. After being mixed, the absorbing values were measured at 700 nm, and Vc was used as a positive control.

The total antioxidant capacity (T-AOC) was measured by the following method: 0.2 ml sample was taken in a test tube with 2 ml solution (containing 0.6 mol/l sulfuric acid, 28 mmol/l trisodium phosphate and 4 mmol/l ammonium molybdate) in 95°C water bath for 90 min, and the absorbing values were measured at 695 nm.

The hydroxyl radical scavenging ability was measured by Fenton method [31]. One milliliter ferrous sulfate solution (9 mmol/l), 1 ml of salicylic acid ethanol solution (9 mmol/l), 1 ml of BGP and 1 ml of hydrogen peroxide solution (8.8 mmol/l) were uniformly mixed in a test tube. After 37 min in 37°C water bath, the cells were centrifuged at 6000 r/min for 10 min and the supernatant was measured for absorbance at 510 nm, and Vc was used as a positive control. The hydroxyl radical scavenging rate (SR) was calculated according to the following formula: SR (%) = ((A − A_0_)/A_0_) × 100, where A_0_, the absorbance of the blank control and A, the absorbance of the sample.

The scavenging capacity of 2,2′-azino-bis (3-ethylbenzenthiazoline-6-sulfonic) acid radical (ABTS^+^) was measured as follows: ABTS solution (7 mmol/l) and potassium persulfate solution (4.9 mmol/l) were mixed in equal volume, and placed in the dark for 20 h to obtain ABTS stock solution, which was diluted to give an absorbance of 0.7 at 734 nm. A total of 0.8 ml ABTS and 0.2 ml sample was mixed to avoid light for 6 min at room temperature, and absorbing values were measured at 734 nm. Meanwhile, Vc was used as a positive control. The ABTS radical SR was calculated according to the following formula: SR (%) = (1 − (A − A_0_)/A_0_) × 100, where A_0_ stands for distilled water and A stands for the sample.

Scavenging ability of DPPH free radical were measured as follows: 2 ml polysaccharide was placed in a test tube and 2 ml DPPH in ethanol solution (0.2 mmol/l) was added, and mixed well to react for 30 min in dark. The absorbing values were measured at 517 nm, and Vc was used as a positive control. The DPPH radical SR was calculated according to the following formula: SR (%) = (1 − (A − A_0_)/A_0_ × 100, where A was the absorbing value of the mixture of sample solution and 2 ml of DPPH ethanol solution and A_0_ was the absorbing values of the mixture of water and 2 ml of DPPH ethanol solution.

### Extraction of volatiles

Volatiles were extracted using steam distillation according to a previous report [32]. Thirty gram of wild garlic was ground with a mortar, and put into a 1000-ml round bottom flask at the end of the extraction device and 500 ml distilled water was added, and heated with a temperature-controlled electric heating sleeve. The other end was a 100-ml concentrated bottle containing ethanol, and after the end of the distillation extraction, it was concentrated to 1.0 ml, and subjected to GC-MS analysis.

### GC-MS analysis

The following chromatographic conditions were used: column: HP-5MS (60 m × 0.25 mm ID, 0.25 μm film), inlet temperature: 250°C; injection volume, 10 μl; split ratio, 4:1; carrier gas, He 1.0 ml/min; 50°C (2 min) and 220°C (30 min). The following condition of mass spectrometry was used: transmission line temperature, 240°C; EI source electron energy, 70 eV; electron multiplier voltage, 1635 V; mass scan range, 0–450 amu; ion source temperature, 230°C and quadrupole temperature, 150°C. The results of GC-MS detection were manually analyzed and compared with standard mass spectra to determine the chemical structure of each fraction.

### Participants

All procedures were approved by Human Research Ethics Committee of the Second Hospital of Jilin University (Approval Number JLUS-2017-04-12XY) according to World Medical Association Declaration of Helsinki and the registered clinical number was ChiCTR1900022115 (http://www.chictr.org.cn/historyversionpuben.aspx?regno=ChiCTR1900022115). Informed written consent was obtained from all patients before the present experiment. From September 2015 to June 2016, a total of 324 pregnancies were screened for gestational diabetes at the Department of Gynecology and Obstetrics, The Second Hospital of Jilin University. Socio-demographics were investigated by statistical staff, including gender, age, annual income, education level (primary, college, university (undergraduate), Masters/Ph.D.), race and lifestyle (nutrition intake, physical activity, working habits, smoking and alcohol consumption). Anthropometric measures included height, weight, BMI and waist body circumference.

### Blood glucose assessment

Oral glucose tolerance test (OGTT) was performed at 24–28 weeks of gestation. The test could be carried out earlier if the women had a high glucose level in urine during their prenatal visits, or had a high risk for GDM [33]. Pregnant women were fasted for 8 h before OGTT to avoid strenuous activity and mental stimulation. The fasting blood was collected at 7 a.m. and FBG was measured using DEXTER-Z II (Bayer Medical Co., Ltd., Leverkusen, Germany). Seventy five grams of glucose solution (200 ml) was taken within 5 min. No food, smoking, coffee and tea were allowed during the trial. Food intake would affect glucose tolerance [34]. Women who smoked had higher 1-h blood glucose (1hBG) than non-smokers, which also affected OGTT [35]. Coffee [36] and tea [37] could improve glucose tolerance and impair the test too. Venous blood was taken after 1 and 2 h after started eating a meal, and 1hBG and 2-h blood glucose (2hBG) after OGTT was measured using DEXTER-Z II.

### GDM diagnostic criteria

According to the diagnostic criteria for GDM recommended by the International Association of Diabetes and Pregnancy Group (IADPSG), one of the following criteria was diagnosed as GDM: FBG ≥ 5.1 mmol/l; 75 g OGTT 1hBG ≥ 10.0 mmol/l and 75 g OGTT 2hBG ≥ 8.5 mmol/l [38].

### Dietary survey

A 24-h dietary review questionnaire was performed in all pregnant women to investigate dietary intake during the first and the third trimesters. The 24-h dietary questionnaire of the control and the intervention groups was used to calculate the intake of each nutrient according to ‘Chinese Food Composition Table 2014’ (https://wenku.baidu.com/view/4e3081ceac51f01dc281e53a580216fc700a533c.html).

### Inclusion criteria

First prenatal visit was before 12 weeks’ gestation. All patients were singleton pregnancy and met the diagnostic criteria for GDW. FBG levels were more than 5.1 mmol/l and/or more than 8.5 mmol/l after 2 h. The patients were informed about the content of the present study and volunteered to participate in the present study.

### Exclusion criteria

The patients suffered from hypertension, kidney and or cardiovascular disease. They took the medications that may interfere with sugar and lipid metabolism during pregnancy (e.g. indomethacin, phentolamine, diuretics, phenytoin sodium and pine etc.). Also, the patients taking specified antioxidant were excluded. The patients had placenta previa, threatened abortion and artificial infertility. The patients were lost in the course of the study. Some patients had history of previous adverse pregnancy: unexplained stillbirth or fetus stopped developing.

### Trial design

The present study was a parallel randomized controlled trial with double blind parallel designs. All patients were divided into two groups with equal allocation.

### Patients grouping

**Figure 1:**
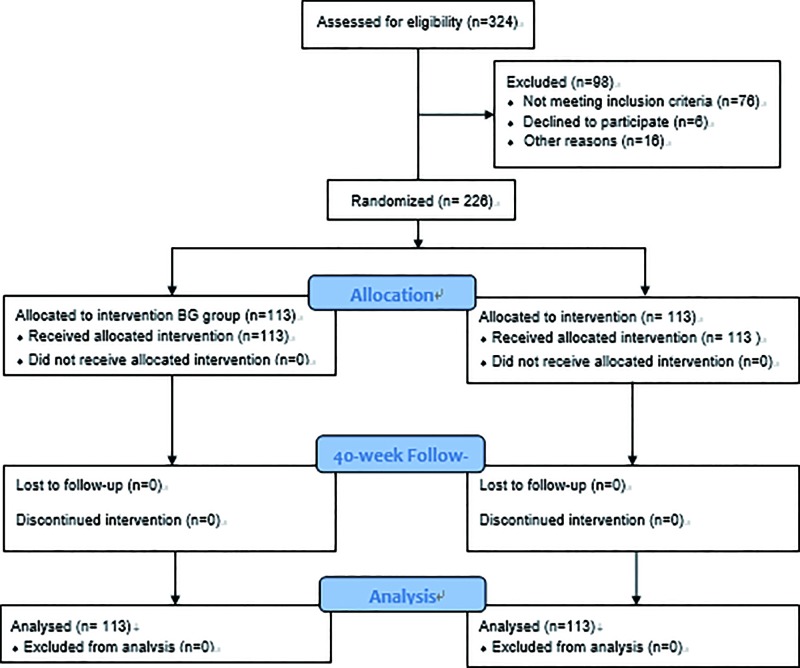
The flowchart of the present study

Sample size was determined by using a power test with the expected power of 0.9 and α of 0.5 [39], and required population size of 220 GDM patients with 110 for each group. Of these pregnant women, 324 patients were recruited. GDM experts enrolled all participants. After inclusion and exclusion criteria, 98 patients were excluded, including hypertension (30 cases), kidney disease (8 cases), cardiovascular disease (15 cases), medications that interfered with sugar and lipid metabolism during pregnancy (23 cases), placenta previa (2 cases), threatened abortion (3 cases), artificial infertility (3 cases), lost in the course of the study (6 cases), history of previous adverse pregnancy (3 cases), unexplained stillbirth (2 cases) and fetus stopped developing (3 cases). Finally, 226 patients were selected to participate in the trial (Figure 1). A statistician generated the random allocation sequence by using a random number table created by a computer and the sequence was concealed until interventions were assigned. The present study was parallel randomized controlled trial and the patients were randomly and evenly assigned into the BG and CG groups. The allocation ratio was 1:1 in a trial comparing two different treatments.

### Intervention

A third investigator (C.D.) assigned participants to interventions after signed informed consent was obtained from each participant. In the BG group, the patients received 5 g black garlic fermented by *L. bulgaricus* daily, and in the CG group, the patients received the black garlic without the fermentation with *L. bulgaricus* daily at home at 11 a.m. in Changchun city (Figure 1). The present study was performed in a double-blind trial, and the investigator and trial personnel, as well as the patients and care providers, were blinded to treatment identity after assignment to interventions. The similar intervention was that black garlic was used in both groups. The treatment duration was 40 weeks from July 2016 to April 2017.

### Primary outcome analysis

The primary outcome was the proportion of patients who were screened for GDM with an OGTT within 4 weeks after the study start date. FBG was measured at 7:30 a.m. and 1hBG and 2hBG were measured exactly 1 and 2 h after start of eating a meal on the same day. All blood glucose was measured by using was measured by using DEXTER-Z II (Bayer Medical Co., Ltd., Leverkusen, Germany). The blood glucose was recorded every 4 days.

### Secondary outcome analysis

Secondary outcomes were from after 4-week primary outcome analysis to the end of the present experiment. Before the end of the present experiment, pregnancy and fetal perinatal outcomes were investigated: (1) pregnancy outcomes in the women included gestational age at delivery, weight gain during pregnancy, presence of induced labor, cesarean section, and preeclampsia, and measured by pregnancy nursing experts; (2) fetal perinatal outcome included whether there was over-production, stillbirth, neonatal death, low birth weight infants, macrosomia, preterm infants, respiratory distress syndrome, hyperbilirubinemia and/or neonatal intensive care unit (NICU), and measured by perinatal nursing experts.

### Measurement of antioxidant activities in GDM patients

Five milliliters of blood was drawn from each patient arm veins with a catheter on the seventh morning of hospitalization within 7–8 a.m. in a stable state. The heparin anticoagulant tube was used to collect 4–5 ml peripheral blood during fasting. The blood sample was centrifuged at 1500 rpm for 10 min. The upper plasma was collected and placed in four centrifuge tubes, and placed in −20°C refrigerator. Malondialdehyde (MDA), superoxide dismutase (SOD), glutathione peroxidase (GSH-PX) and T-AOC kits were purchased from Jiancheng Co. Ltd. (Nanjing, China). Plasma levels of MDA, SOD, GSH-PX and T-AOC were measured using corresponding kits. MDA, SOD, GSH-PX and T-AOC was measured spectrophotometrically on a UV-Vis spectrophotometer (Hitachi, Japan) at 535, 560, 340 and 520 nm according to manufacturer’s instructions, respectively.

### Measurement of side effects

The side effects of garlic included unpleasant taste (100%), halitosis (90%) and nausea (30%) [40] and burning sensations [41], but no side effect for black garlic was reported. *L. bulgaricus* may cause hives, chest tightness, difficulty in breathing, and swelling of face, lips, tongue and/or throat. All these side effects were investigated during the whole period of the present experiment.

### Statistical analysis

The data were presented as mean values ± S.D. (standard deviation). Statistical analysis was performed using the SPSS 20.0 statistical software package (IBM-SPSS, Armonk, NY, U.S.A.). A Chi-square test was used to compare count data for primary and secondary outcomes between two groups. The *t* test (for normal distribution) or Mann–Whitney U test was used to compare measurement data for primary and secondary outcomes between two groups. The statistical difference was significant if *P*<0.05.

## Results

### Main ingredients in black garlic

The changes in main ingredients between fresh garlic and black garlic are provided in (Table 1). Before fermentation, the statistical difference for all parameters was insignificant between two groups (Table 1, *P*>0.05). The flavor was softened in black garlic after fermentation and sweetness developed. The sugar contents increased in black garlic when compared with fresh garlic (Table 1, *P*<0.05). The reducing sugar reached the highest level in the black garlic fermented with *L. bulgaricus*. The lipid contents were reduced in the BG group when compared with the CG group (Table 1, *P*<0.05).

**Table 1:** The comparison of the main ingredients (g) of garlic (100 g) between two groups

	Before fermentation		After fermentation	
	BG	CG	BG	CG
Water (g)	63.8	62.3	53.7	52.5
Total sugar	7.2	7.4	28.5	26.1
Reducing sugar	0.3	0.3	9.7	6.4
Polysaccharide	6.9	7.1	18.8	19.7
Protein	5.2	5.5	7.4	6.9
Lipid	10.2	9.8	4.6	5.2

### *L. bulgaricus* increased the contents of *glucofuranoside* in black garlic

**Figure 2:**
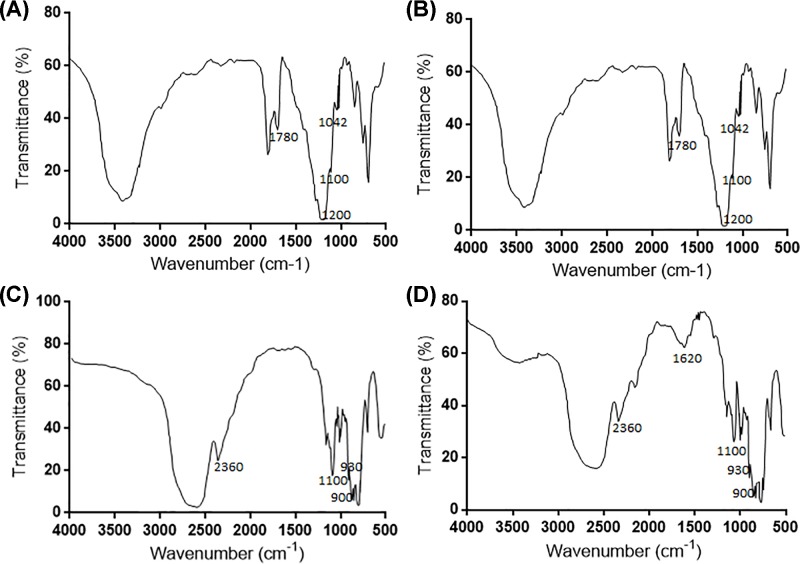
FITR analysis of the functional groups in the polysaccharide of garlic (**A**) The polysaccharide in BG group before fermentation. (**B**) The polysaccharide in CG group before fermentation. (**C**) The polysaccharide in BG group after fermentation. (**D**) The polysaccharide in CG group after fermentation. FITR; Fourier Transform infrared.

FTIR analysis showed that a significant absorption region from 3150 to 3750 cm^−1^ for OH stretching frequencies [42] in fresh garlic (Figure 2A,B,D) and an absorption region from 2450 to 3050 cm^−1^ for CH stretching frequencies [43] in black garlic (Figure 2C,D). The results suggested that there were some polysaccharides in the black garlic, and also could be found in the fresh garlic. *L. bulgaricus* promoted the transformation of the polysaccharides in fresh garlic. There was the spectra of glycosides of uronic acids at 1780 cm^−1^ [44] in fresh garlic (Figure 2A,B). The absorbing values at 1200 and 1100 cm^−1^ are both assigned to the coupling of CO [45] and CC [46] stretching frequencies (Figure 2A,B). Absorbance at 930 cm^−1^ was COH deformation [47] and 900 cm^−1^ was CH bending region [48] (Figure 2C,D). The present findings approved that the garlic was with absorbing peaks of polysaccharides.

**Figure 3:**
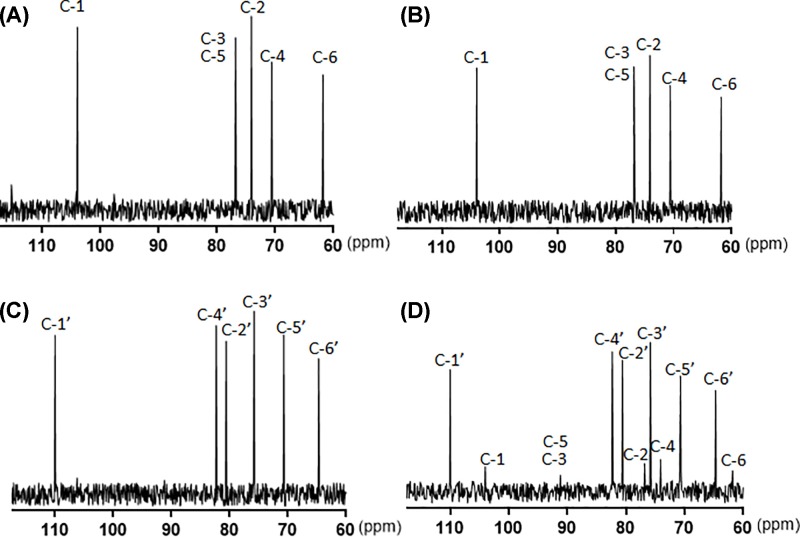
^13^C NMR analysis of the polysaccharide structure of garlic (**A**) The polysaccharide in BG group before fermentation. (**B**) The polysaccharide in CG group before fermentation. (**C**) The polysaccharide in BG group after fermentation. (**D**) The polysaccharide in CG group after fermentation.

^13^C NMR analysis indicated that there were four peaks in BGP spectra. The peaks at 104.8, 77.5, 77.5, 73.6 and 70.2 ppm were C-1, C-3, C-5, C-2 and C4 in polysaccharides in both the groups before fermentation [49], respectively. After the fermentation, the difference might be caused by the different sugar moiety. Before fermentation, the peaks were 104.0, 76.8, 74.1, 70.6 and 61.8 ppm in both the groups (Figure 3A,B), which were compatible to β-d-glucopyranoside [50]. After fermentation, the peaks were 110.0, 82.3, 80.6, 75.8, 70.7 and 64.7 ppm in the black garlic fermented with probiotics (Figure 3C), which was compatible to β-d-glucofuranoside [51], whereas there were the peaks 104.0, 76.8, 74.1, 70.6, 61.8, 110.0, 82.3, 80.6, 75.8, 70.7 and 64.7 ppm with β-d-glucopyranoside and β-d-glucofuranoside in the control group (Figure 3D). The present findings proved that *L. bulgaricus* promoted the transformation of the glucopyranoside in fresh garlic to glucofuranoside in black garlic.

### *L. bulgaricus* increased antioxidant capacities of black garlic

**Figure 4:**
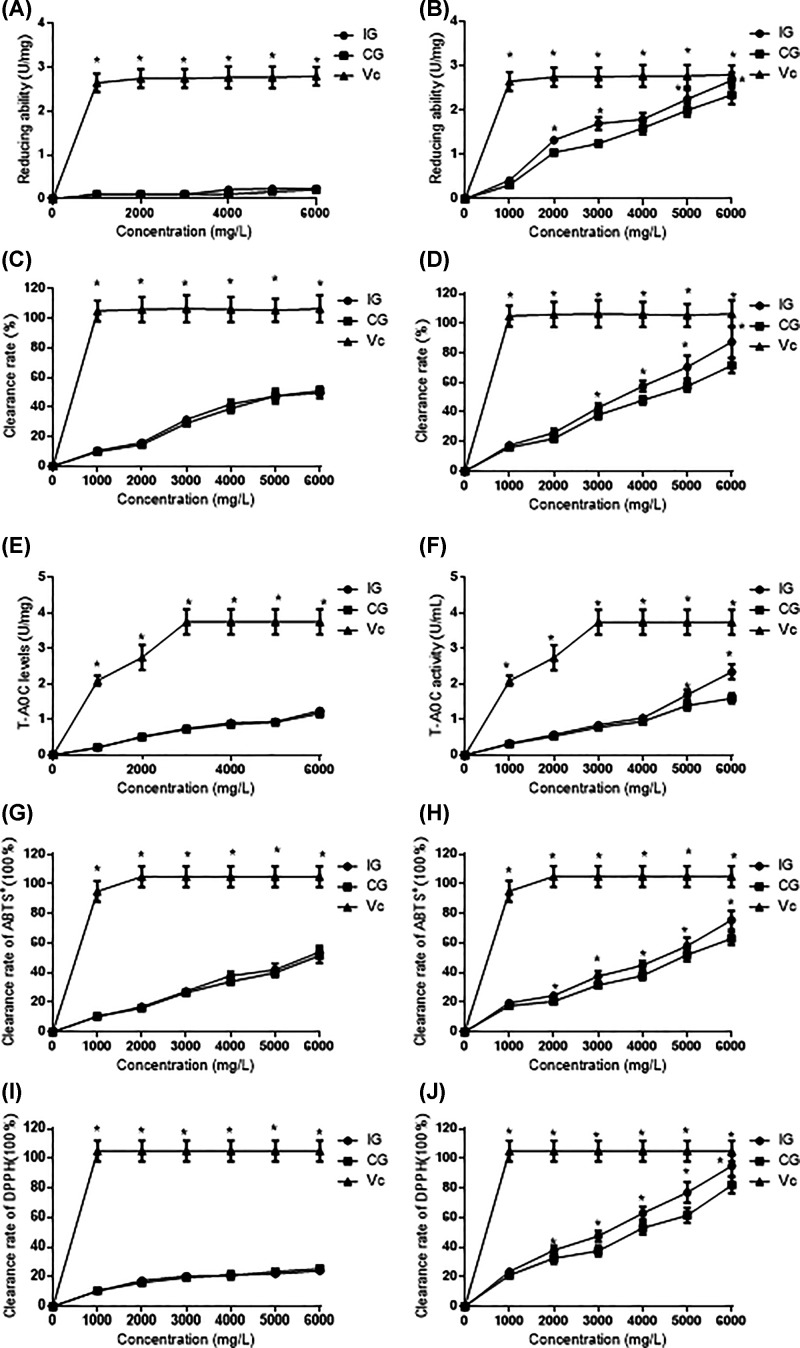
The comparison of antioxidant capacity (**A**) The reducing ability of polysaccharide among BG, CG and Vc groups before fermentation. (**B**) The reducing ability of polysaccharide among BG, CG and Vc groups after fermentation. (**C**) The T-AOC of polysaccharide among BG, CG and Vc groups before fermentation. (**D**) T-AOC of polysaccharide among BG, CG and Vc groups after fermentation. (**E**) The hydroxyl radical scavenging ability of polysaccharide among BG, CG and Vc groups before fermentation. (**F**) Hydroxyl radical scavenging ability of polysaccharide among BG, CG and Vc groups after fermentation. (**G**) ABTS free radical scavenging ability of polysaccharide among BG, CG and Vc groups before fermentation. (**H**) ABTS free radical scavenging ability of polysaccharide among BG, CG and Vc groups after fermentation. (**I**) DPPH free radical scavenging ability of polysaccharide among BG, CG and Vc groups before fermentation. (**J**) DPPH free radical scavenging ability of polysaccharide among BG, CG and Vc groups after fermentation. *P<0.05 vs. a control group.

Some polysaccharides with strong reducing ability can provide electrons and free radical reactions, which interrupt the free radical reaction chain, and prevent the formation of lipid peroxidation products. Figure 4A showed that the reducing ability of polysaccharides was similar between two groups (*P*>0.05) and lower than Vc group (*P*<0.05) before fermentation. The reducing ability of the BGP gradually increased with *L. bulgaricus* after fermentation and exhibited significant dose-dependent relationship (*P*<0.05, Figure 4B). T-AOC was similar between two groups (*P*>0.05) and lower than Vc group (*P*<0.05, Figure 4C) before fermentation. The T-AOC of the BGP gradually increased with *L. bulgaricus* after fermentation and exhibited dose-dependent relationship (*P*<0.05, Figure 4D).

Hydroxyl radicals are very reactive and have strong electron-accepting ability. They can react with many biomacromolecules and are harmful and destructive reactive oxygen free radicals. The hydroxyl radical scavenging ability was similar between two groups (*P*>0.05) and lower than the Vc group (*P*<0.05, Figure 4E) before fermentation. The hydroxyl radical scavenging ability of the BGP gradually increased with *L. bulgaricus* after fermentation and exhibited significant dose–effect relationship (*P*<0.05, Figure 4F).

The ABTS method is a widely used method for indirect detection of antioxidants. ABTS free radical scavenging ability was similar between two groups (*P*>0.05) and lower than the Vc group (*P*<0.05, Figure 4G) before fermentation. ABTS free radical scavenging ability of the BGP gradually increased with the addition of *L. bulgaricus* after fermentation and exhibited significant dose–effect relationship (*P*<0.05, Figure 4H).

DPPH is widely used in the evaluation of antioxidants *in vitro* and a very stable nitrogen-centered free radical. Its ethanol solution is blue-violet and has strong absorption at 517 nm. When an antioxidant is added, blue-violet will change. It is light yellow or colorless, and the absorbance is reduced. DPPH free radical scavenging ability was similar between two groups (*P*>0.05) and lower than the Vc group (*P*<0.05, Figure 4I) before fermentation. The scavenging ability of DPPH of the BGP gradually increased with the addition of *L. bulgaricus* after fermentation and exhibited significant dose-dependent relationship (*P*<0.05, Figure 4J).

### The main volatiles of fresh garlic and black garlic

Table 2 showed that there were 28 volatile components with higher relative content of garlic in the dormant period, and the highest content of these compounds was diallyl disulfide, allyl formate, diallyl trisulfide, alkene, propyl sulfide and tetrahydro-2-ethylthiophene. There were no significant statistical differences between two groups (*P*>0.05).

**Table 2:** Garlic volatile components in two groups

Peaks	Compounds	Molecular formula	Molecular weight	Retention time (min)	Relative percent (%)	
					CG	IG
1	Allyl formate	C_4_H_6_O_2_	86.08	5.46	17.86	17.51
2	Allyl sulfide	C_6_H_1_OS	114.20	6.37	6.16	5.98
3	N-octylpropyl sulfide	C_4_H_10_S	90.18	9.33	0.17	0.18
4	2-methylpyrazine	C_5_H_6_N_2_	94.11	9.58	0.19	0.17
5	Allyl methyl disulfide	C_4_H_8_S_2_	120.24	9.94	1.59	1.62
8	Dimethyltrisulfide	C_2_H_6_S_3_	126.26	12.81	1.42	1.45
10	A-angelica lactone	C_5_H_6_O_2_	98.10	14.47	0.61	0.59
12	N,NW-methine triformamide	C_4_H_7_N_3_O_3_	145.12	15.06	0.72	0.70
13	Diallyl disulfide	C_8_H_12_O_4_	172.18	15.46	25.41	25.04
14	Furfural	C_5_H_4_O_2_	96.08	15.89	3.84	3.76
15	2-acetyl scent	C_6_H_6_O_2_	110.11	16.66	1.09	1.11
17	Dimethyl malate	C_6_H_10_O_5_	162.14	17.26	1.61	1.52
22	5-methylfurfural	C_6_H_6_O_2_	110.11	18.71	2.30	2.37
23	Diallyl trisulfide	C_6_H_10_S_3_	178.34	19.08	15.52	15.64
24	2,2-dimethyl-1,3-dithiane	C_6_H_12_S_2_	148.00	19.45	2.13	2.21
25	Y-butyrolactone	C_4_H_8_O_4_	120.10	20.19	0.51	0.54
28	Sterol	C_5_H_6_O_2_	98.10	21.15	0.97	0.92
30	Y-caprolactone	C_6_H_10_O_2_	114.14	22.29	0.25	0.23
31	3-ethyl-3-hexanol	C_8_H_18_O	130.23	22.88	0.19	0.18
33	2-Methoxycyclohexa-2,5-diene-1,4-dione	C_7_H_6_O_3_	138.12	23.17	2.42	2.39
35	146-trimethylnaphthalene	C_13_H_14_	170.25	24.59	2.12	2.14
36	1-methyl-4-piperidone-3 methyl decanoate	C_8_H_13_NO_3_	171.19	25.69	0.19	0.18
38	3-vinyl-3,4-dihydro-1,2-dithiocyclohexene	C_6_H_8_S_2_	144.26	25.93	0.39	0.36
42	1,3,5-trithiane	C_3_H_6_S_3_	138.27	28.49	0.19	0.20
52	Methyl 14-methylpentadecanoate	C_17_H_34_O_2_	270.45	35.33	0.51	0.53
56	2-ethyltetrahydrothiophene	C_6_H_12_S	116.22	38.92	4.81	4.84
62	4-methyl-1-carbaldehyde	C_12_H_10_O	170.20	43.93	0.38	0.41
65	Dibutyl phthalate	C_16_H_22_O_4_	278.34	44.99	0.93	0.95

Table 3 showed that the main volatile components in fermented black garlic were 29 and 27 kinds of compounds in BG and CG, respectively, and the high-level volatile components were thiane, diallyl disulfide, 2-ethyltetrahydrothiophene, 2-vinyl-1,3 dithiane, N, N″-dimethylthiourea etc. In addition to the pungent odor, diallyl trisulfide has burning and sulfur odor, and the irritating odor was significantly reduced after garlic fermentation. The content of thiophene was significantly increased compared with fresh garlic, which produced light fragrance in black garlic. Experiments showed that the irritating odor of garlic was greatly reduced after high temperature fermentation, and the aroma was increased. The total amount of volatile sulfur in black garlic inhibited the synthesis of carcinogenic nitrosamines, the formation and growth of cancer cells, lowered blood pressure and prevented cardiovascular and cerebrovascular diseases. Other low molecular volatile compounds (especially allyl thiol and hydrogen sulfide) subsequently promoted the formation of dipropylene sulfide and cyclic compounds, such as -allyl-thioether and 2-vinyl-1,3-thiane. On the other hand, allyl alcohol also combined with allyl sulfide, alkene propyl mercaptan or alliin/deoxy-aromatic acid to form diallyl sulfide. High-temperature fermentation of garlic significantly improved the pungent odor of fresh garlic and produce a large number of sulfur-containing compounds that were beneficial to the health of GDM patients. They were 2-methyl-1-butanol, linalool, and ethyl acetate in IG group (Table 3). *L. bulgaricus* increased the aroma smell of black garlic and produced more ingredients.

**Table 3:** Volatile components of black garlic between two groups

Peaks	Compounds	Molecular formula	Molecular weight	Retention time (min)	Relative percent (%)	
					IG	CG
**1**	Acryl alcohol	C_3_H_6_O	58.08	5.49	1.69	1.64
**2**	Allyl sulfide	C_6_H_10_S	114.21	6.39	0.81	0.83
**3**	Ethanol	C_2_H_5_O_6_	46.07	7.13	2.98	
**4**	2-methyl-1-butanol	C_5_H_12_O	88.15	8.08	0.76	
**6**	Linalool	C_10_H_18_O	154.25	9.21	2.03	
**7**	Allyl methyl disulfide	C_4_H_8_S_2_	120.24	9.97	4.54	4.73
**8**	1,3-dithiane	C_4_H_8_S_2_	120.24	10.18	1.10	1.17
**10**	Citronellol	C_10_H_20_O	156.27	11.38	0.95	
**11**	Dimethyltrisulfide	C_2_H_6_S_4_	126.26	12.96	0.69	0.71
**14**	Tetrahydro-2-hydrogen-1,4,6-oxodidiazepine ring	C_5_H_10_N_2_OS	146.00	15.51	5.04	5.49
**15**	Diallyl disulfide	C_8_H_12_O_4_	172.18	16.11	15.08	17.53
**16**	2-vinyl-1,3-dithiane	C_6_H_10_S_2_	146.00	16.27	8.90	8.81
**17**	NN-dimethylthiourea	C_3_H_8_N_2_S	104.17	17.21	8.12	8.00
**18**	Ethylthiourea	C_3_H_8_N_2_OS	120.17	17.73		0.05
**19**	2-ethyltetrahydrothiophene	C_6_H_12_S	116.22	19.23	11.10	13.24
**20**	(methylthio)acetonitrile	C_3_H_5_NS	87.14	19.32	1.32	1.39
**21**	H1-propenyl-1 cyano)-butane	C_7_HUS	118.00	20.84	1.41	1.36
**23**	5-methyl-1,2,4-triazole-3 sterol	C_3_H_5_N_3_S	115.16	23.02	0.41	0.49
**24**	3-vinyl-3,4-dihydro-1,2-dithiazide	C_6_H_8_S_2_	144.26	23.16	3.65	3.87
**25**	2-ethylidene-1,3-dithiane	C_6_H_10_S_2_	146.00	23.88		0.14
**26**	3-vinyl-3,4-dihydro-1,2-dithiane	C_6_H_8_S_2_	144.26	24.79	16.69	17.56
**28**	3,5-diethyl-1,2,4-tritetrahydrothiophene	C_6_H_12_S_3_	180.35	25.18	0.09	0.08
**29**	135-trithiane	C_3_H_6_S_3_	138.27	25.72	0.16	0.18
**30**	(2 yl thio)-acetonitrile	C_5_H_7_NS	113.00	25.78	0.12	0.11
**31**	Diallyl trisulfide	C_6_H_10_S_3_	178.34	25.99	2.87	2.95
**32**	135-trithiane	C_3_H_6_S_3_	138.27	27.34		0.06
**33**	2-n-propylthiophene	C_7_H10S	126.22	27.61	0.08	0.07
**34**	(2 yl thio)-acetonitrile	C_5_H_7_NS	113.00	28.03	0.09	0.10
**37**	135-trithiane	C_3_H_6_S_3_	138.27	29.89	0.15	0.14
**38**	12-dithiocyclopentane	C_3_H_6_S_2_	106.21	30.74		0.08
**41**	Ethyl acetate	C_4_H_8_O_2_	88.11	33.65	2.14	
**46**	Propyl propanoate	C_6_H_12_O_2_	116.16	35.72	0.19	
**54**	2-thiophene	C_4_H_3_NO_2_S	129.14	42.68	1.14	1.15

### Clinical characteristics

After the selection of inclusion and exclusion criteria, 226 GDM patients participated in the present experiment and 98 GDM patients were excluded (Figure 1). Among 226 GDM patients, the proportion of pre-pregnancy BMI ≥ 24 kg/m^2^ was 43.5%; age ≥ 35 years was 37.6% and PCOS history was 19.5%. The history of large childbirth was 8.8% and the family history of diabetes was 31.9%. There was no significant difference in demographic variables between the control and intervention groups (Table 4, *P*>0.05).

**Table 4:** Baseline characteristics of GDM patients between two groups

	*L. bulgaricus*	Control group	t or χ^2^	*P*-values
Age, year	34.32 ± 6.47	34.51 ± 6.56	−0.63	0.52
Prepregnancy weight (kg)	58.48 ± 7.36	58.17 ± 7.85	0.45	0.68
Prepregnancy BMI (kg/m^2^)	24.18 ± 3.91	23.72 ± 3.86	0.34	0.73
Waist circumstance (cm)	88.94 ± 9.38	90.53 ±10.12	0.45	0.52
Gestational weeks	12.14 ± 2.46	11.98 ± 2.27	0.67	0.39
**Physical activity**				
Walking	51 (45.13)	48 (42.48)	0.74	0.95
Jogging	19 (16.81)	22 (19.47)		
Swimming	8 (7.08)	9 (7.96)		
Sports	10 (8.85)	12 (10.62)		
Yoga	25 (22.12)	22 (19.47)		
Smoking	26 (23.01)	30 (26.55)	0.38	0.54
Drinking	40 (35.4)	34 (30.09)	0.72	0.40
FBG, mmol/l	5.51±0.37	5.53 ± 0.42	0.15	0.87
History of PCOS	20 (17.7)	24 (21.24)	0.45	0.50
Family history of diabetes	34 (30.09)	38 (33.63)	0.33	0.57
Large childbirth	8 (7.08)	12 (10.62)	0.88	0.35
Monthly income, RMB	5378 ± 2654	5027 ± 2561	0.73	0.26
**Education level**				
Primary	4 (3.54)	6 (5.31)	3.00	0.56
High school	21 (18.58)	19 (16.81)		
College	25 (22.12)	28 (24.78)		
University	52 (46.02)	55 (48.67)		
Masters/Ph.D.	11(9.73)	5(4.42)		
**Race, *n* (%)**				
Minority	10 (8.85)	14 (12.39)	0.75	0.39
Han people	103 (91.15)	99 (87.61)		

*n*=113 for each group.

### The comparison of nutrition intake between two groups

Dietary energy [52] and nutrient intake [53] have been reported to be associated with GDM risk or progression. Compared with the control group, the actual intake of nutrients was similar to that of the interventional group, including energy, proteins, carbohydrates, vitamin A, folic acid, calcium, iron and zinc in the early pregnancy (Table 5, *P*>0.05).

**Table 5:** The comparison of nutrition comparison between two groups

	*L. bulgaricus* group	Control group	*t* values	*P*-values
**Early stage**				
**Energy (kcal)**	1290 ± 351	1265 ± 363	1.64	0.67
**Protein (g)**	36.6 ± 13.8	37.1 ± 14.2	1.35	0.48
**Fat (g)**	37.8 ± 19.2	36.9 ± 19.5	1.56	0.35
**Carbohydrate (g)**	145.9 ± 58.8	152.9 ± 61.3	3.81	0.56
**Vitamin A (μg)**	431.6 ± 306.9	429.6 ± 321.1	0.26	0.84
**Vitamin C (mg)**	93.1 ± 56.7	92.8 ± 55.8	0.35	0.62
**Folic acid (mg)**	213.4 ± 161.0	223.5 ± 156.4	1.49	0.27
**Calcium (mg)**	405.6 ± 341.7	412.5 ± 336.4	1.71	0.58
**Iron (mg)**	17.8 ± 7.8	18.2 ± 7.5	0.64	0.91
**Zinc (mg)**	6.8 ± 2.1	6.9 ± 2.4	0.24	0.95
**Middle and late stages**				
**Energy (kcal)**	1293 ± 356	1328 ± 362	0.17	0.89
**Protein (g)**	36.7 ± 13.8	35.5 ± 14.1	0.20	0.74
**Fat (g)**	36.4 ± 19.7	37.2 ± 20.3	0.83	0.60
**Carbohydrate (g)**	146.3 ± 58.4	142.5 ± 56.9	0.35	0.82
**Vitamin A (μg)**	411.8 ± 304.1	406.2 ± 313.6	0.54	0.67
**Vitamin C (mg)**	92.3 ± 56.2	93.7 ± 55.9	0.39	0.84
**Folic acid (mg)**	211.5 ± 169.2	208.3 ± 172.6	0.28	0.73
**Calcium (mg)**	408.6 ± 341.8	412.5 ± 336.2	0.41	0.52
**Iron (mg)**	18.5 ± 7.3	19.1 ± 8.0	0.76	0.40
**Zinc (mg)**	6.9 ± 2.9	7.1 ± 2.6	2.21	0.59

*n*=113 for each group.

### *L. bulgaricus* could not affect blood glucose levels of primary outcomes

No one withdrew from the present experiment and the GDM number was same for the subjects who were randomly assigned, received intended treatment and analyzed for the primary outcome between two groups. Blood glucose monitoring is a scalable and practical method to prevent GDM progression [54]. After 1-month treatment, FBG, 1hBG and 2hBG levels in the CG group were similar with the BG group (Table 6, *P*>0.05). FBG, 1hBG and 2hBG difference for the observed coverage of the 95% confidence interval was from 0.25 to 0.73 mmol/l, 0.5 to 1.1 mmol/l and 0.5 to 1.3 mmol/l mg/dl (*P*>0.05). These results suggested that *L. bulgaricus* could not affect blood glucose level and glucose tolerance of primary outcomes in a short term.

**Table 6:** The primary outcome of the number of abnormal OGTT values between two groups

	Abnormal OGTT		
	FBG	1hBG	2hBG
**Control group**	78 (69.03)	45 (39.82)	66 (58.41)
***L. bulgaricus* group**	82 (72.57)	40 (35.4)	68 (60.18)
**χ^2^**	0.342	0.471	0.073
***P***	0.558	0.492	0.787

*n*=113 for each group.

### *L. bulgaricus* reduced blood glucose levels of secondary outcomes

After 24- and 28-week treatment, no one withdrew from the present experiment. FBG, 1hBG and 2hBG levels in the CG group were higher than in the BG group (Table 7, *P*<0.01 for 1hBG and *P*<0.001 for FBG and 2hBG). These results suggested that *L. bulgaricus* reduced blood glucose level and increased glucose tolerance. FBG, 1hBG and 2hBG difference for the observed coverage of the 95% confidence interval was from 1.5 to 3.3 mmol/l, 1.1 to 2.5 mmol/l and 1.6 to 3.8 mmol/l (*P*<0.01).

**Table 7:** The secondary outcome of the number of abnormal OGTT values between two groups

	Abnormal OGTT		
	FBG	1hBG	2hBG
**Control group**	89 (78.76)	52 (46.02)	76 (67.26)
***L. bulgaricus* group**	65 (57.52)	32 (28.32)	48 (42.48)
**χ^2^**	11.740	7.579	14.009
***P***	0.000^†^	0.006*	0.000^†^

*n*=113 for each group. *P<0.01. ^†^*P*<0.001 *vs.* control group.

### *L. bulgaricus* improved pregnancy outcomes between two groups

The weight gain of the intervention group was lower than that of the control group during the gestational period from 36.9 to 39.9 weeks (Table 8, *P*<0.05). Cesarean section and labor induction rate, preeclampsia incidence was not statistically significant between two groups (Table 8, *P*>0.05). *L. bulgaricus* improved pregnancy outcomes between two groups by controlling weight gain and weight difference for the observed coverage of the 95% confidence interval that was from 1.65 to 3.89 kg between two groups.

**Table 8:** Pregnancy outcomes between two groups

	Gestational weeks	Weight gain (kg)	Cesarean section, *n* (%)	Labor induction, *n* (%)	Preeclampsia, *n* (%)
**Control group**	38.12 ± 1.82	16.47 ± 5.62	23 (20.35)	0 (0.00)	2 (1.77)
***L. bulgaricus* group**	37.84 ± 0.94	13.85 ± 4.83	26 (23.01)	0 (0.00)	0 (0.00)
**t**	0.63	2.68	0.24	0.00	0.50
***P***	0.28	*0.03	0.68	1.00	0.48

*n*=113 for each group. *P<0.05 *vs.* control group.

### *L. bulgaricus* reduced the incidence of perinatal complications

The incidence of huge childbirth, premature baby and NICU was lower in the intervention group than that in the control group (Table 9, *P*<0.05). There was no significant difference for other perinatal complications between the two groups (Table 9, *P*>0.05).

**Table 9:** The incidence of perinatal complications between both groups

Groups	Control group	*L. bulgaricus* group	χ^2^	*P*-values
**Overdue production**	0 (0.00)	0 (0.00)	0	1
**Stillbirth**	0 (0.00)	0 (0.00)	0	1
**Newborn death**	0 (0.00)	0 (0.00)	0	1
**Low birth weight**	4 (3.54)	2 (1.77)	0.17	0.68
**Huge childbirth**	16 (14.16)	4 (3.54)	7.90	*0.01
**Premature baby**	14 (12.39)	2 (1.77)	9.69	*0.01
**Respiratory distress syndrome**	10 (8.85)	6 (5.31)	1.08	0.30
**Hyperbilirubinemia**	0 (0.00)	0 (0.00)	0	1
**NICU**	10 (8.85)	2 (1.77)	5.63	*0.02

*n*=113 for each group. *P<0.05 *vs*. control group.

### Comparison of plasma MDA, SOD, T-AOG and GSH-PX between two groups

There was no statistical difference in plasma levels of MDA, SOD, T-AOG and GSH-PX between two groups at admission (Table 10, *P*>0.05). The results suggested the patients’ grouping would not affect the measurement of antioxidant activities. After 40-week therapy, plasma MDA in the CG group (3.65 ± 1.42 nmol/ml) was higher than in the BG group (2.36 ± 1.27 nmol/ml, *P*<0.01), plasma SOD in the CG group (62.89 ± 15.49 U/ml) was lower than in the BG group (79.51 ± 8.53 U/ml), plasma T-AOC in the CG group (6.38 ± 3.06 U/ml, *P*<0.001) was lower than in the BG group (9.14 ± 3.27 U/ml, *P*<0.001), and plasma GSH-PX in the CG group (136.43 ± 48.19 U/ml) was lower than in the BG group (192.39 ± 52.61 U/ml, *P*<0.01, Table 10). The results suggested that *L. bulgaricus* increased antioxidant capacity of GDM patients.

**Table 10:** Comparison of plasma MDA, SOD, T-AOG and GSH-PX between two groups

Parameters	*L. bulgaricus* group	Control group	*t* values	*P*-values
**At admission**				
MDA (nmol/ml)	3.48 ±1.33	3.65 ±1.41	0.547	0. 261
SOD (U/ml)	59.57 ± 9.34	54.64 ±11.32	0.249	0. 091
T-AOC (U/ml)	6.01 ± 3.11	6.36 ± 2.55	0.197	0. 473
GSH-PX (U/ml)	162.39 ± 41.20	150.27 ± 42.03	0.348	0. 161
**After 40-week therapy**				
MDA (nmol/ml)	2.36 ±1.27	3.65 ± 1.42	8.398	0. 003*
SOD (U/ml)	79.51 ± 8.53	62.89 ±15.49	4.562	0. 004*
T-AOC (U/ml)	9.14 ± 3.27	6.38 ± 3.06	6.815	0. 000^†^
GSH-PX (U/ml)	192.39 ± 52.61	136.43 ± 48.19	9.784	0. 002*

*P<0.01. ^†^*P*<0.001 *vs*. control group.

### Side effects

There was no unpleasant taste, halitosis, nausea, burning sensations, garlic odor, hives, chest tightness, difficulty in breathing, and swelling of face, lips, tongue and/or throat caused by black garlic and *L. bulgaricus* in both groups.

## Discussion

*L. bulgaricus* increased the antioxidant capacities of black garlic by scavenging hydroxyl radicals, ABTS and DPPH free radicals. *L. bulgaricus* reduced the levels of FBG and 2hBG, and incidence of perinatal complications (*P*<0.01). There may be other mechanism for the improvement of hyperglycemic state caused by *L. bulgaricus*. A regular consumption of yogurt with *L. bulgaricus* can improve human intestinal microbiota [55]. Diabetic patients had an imbalance in gut microbiota [56], which also caused maternal obesity during pregnancy on offspring metabolism, and probiotic interventions could control the risk of obesity and metabolic diseases [57]. The previous review states that the composition of human gut microbiota would affect hyperglycemic states, which are associated with the diabetes with different severities [58]. That until now, no study exists to suggest that gut microbiota affects GDM. Further work is highly needed to explore the underlying mechanisms and accordingly develop therapeutic strategies. Furthermore, *L. bulgaricus* reduced plasma MDA level and increased SOD, GSH-PX and T-AOC (*P*<0.01). The present findings demonstrated that *L. bulgaricus* improved antioxidant activities of GDM patients.

*L. bulgaricus* improved the polysaccharides of black garlic and further prevented GDM progression. In the OGTT test, the levels of FBG were higher in CG group than in BG group after 1 or 2 h of taking sugar (Table 7). The glycemic control effect in the CG group was lower than in the BG group. GDM patients were mainly manifested as postprandial 2-h hyperglycemia [59]. This may be due to the fact that the postprandial insulin secretion of pregnancies with normal glucose metabolism is earlier than that of GDM patients, and glucose is associated with fast recovery in GDM patients [60,61]. After black garlic fermentation with *L. bulgaricus*, insulin resistance was improved in GDM women. In the control group, the risk of developing GDM increased as the month of pregnancy increased and insulin resistance still worsened. Unlike postprandial blood glucose elevations, FBG elevations are parallel to impaired glucose tolerance. Patients with abnormal FBG are not simply insulin resistant, and may have impaired β-cell damage and insulin secretion.

The effects of black garlic on intestinal microbiome are seldom reported and should be explored in the future work. The extract of black garlic can be used as oral healthcare products and it also has the potential to control oral bacterial infections [62]. The improvement of composition of intestinal microbiota has been reported to reduce blood glucose levels and control body weight gain in the diabetes patients [63]. Improved gut microbes respond to neuroendocrine, and immune biochemical messages, improve hyperglycemic states of host and have health-promoting properties in the therapy of diabetes [58]. Butyrate production of gut microbiota may be one of the important mechanisms in regulating energy metabolism by reducing lipid and glucose levels [64].

Weight gain during pregnancy is a concern for the prevention and treatment of GDM. During pregnancy, body weight gains too fast and fat accumulates, which continuously stimulates the secretion of insulin from pancreatic islet β-cells and triggers hyperinsulinemia. The fatty acid receptors distributed in per unit area of islet cells are relatively reduced, will decrease insulin sensitivity, reduce insulin action, induce excessive secretion of insulin and result in insulin resistance and dysfunction of pancreatic β-cell [65,66]. The present study showed that weight gain in the BG group was lower than the control group (Table 8, *P*<0.05). The weight gain of pregnant women with early intervention was easily controlled within a reasonable range, suggesting it was necessary for early screening, diagnosis and treatment of GDM. Furthermore, women’s dietary intake was studied only in the first and third trimesters but not in the second trimester. Maternal weight change in the first trimester but not the second or the third could affect newborn size [67,68], and thus the first trimester should be selected and the third trimester was selected randomly.

Perinatal period is a critical period of fetal growth and development, and nutrition should be provided for fetal growth and development from the mother. However, long period of maternal hyperglycemic environment will stimulate pancreatic β-cell proliferation, insulin secretion and hyperglycemia on fetus, and cause serious effects on the endocrine system and development of various organs of the fetus [69]. The black garlic fermentation with *L. bulgaricus* for GDM met maternal and fetal nutritional needs, and achieved glycemic control. Huge children are one of the major adverse outcomes of GDM. Insulin has functions of promoting fat and protein synthesis and inhibiting lipolysis. If the fetus is in a hyperinsulinemic environment, the limbs will develop excessively. Some studies showed that the occurrence of GDM increased not only the incidence of macrosomia, but also the prevalence of obesity in adult offspring [70,71]. Compared with normal weight infants, GDM pregnant women produced more serum cholesterol and triglyceride, so the occurrence of macrosomia and GDM lead to changes in lipid metabolism in neonates [72–74]. The present data showed that the incidence of macrosomia and preterm infants was significantly lower in the BG group than that in the CG group (*P*<0.05).

There were some limitations in the present work. There were many ingredients in black garlic and the molecular mechanism for *L. bulgaricus* affecting the ingredients of black garlic remains unknown. *L. bulgaricus* increased the contents of glucofuranoside and reduced the contents of glucopyranoside, which can be used to induce diabetic risk [75] although the effects of glucofuranoside on diabetes remain unclear. *L. bulgaricus* and black garlic has been used widely in China. However, side effects of black garlic fermentation with *L. bulgaricus* remain unclear although *L. bulgaricus* has been used worldwide as functional food. Furthermore, the synergistic effects of the probiotics and black garlic were not explored either. The present study failed to control some confounding variables (obesity and parity) and GDM patients were not stratified based on disease severity. The potential bias may be caused by the selectivity, such as the persons from the same city and garlic was from the same place. Thus, the components of black garlic may vary from different places. Further work is highly demanded to address these important issues.

## Conclusions

The content of total sugar, reducing sugar and polysaccharide in black garlic was higher than in fresh garlic. *L. bulgaricus* promoted the transformation of the *glucopyranoside* in fresh garlic to *glucofuranoside* in black garlic. *L. bulgaricus* increased the antioxidant capacity of the BGP by increasing the abilities of scavenging hydroxyl radicals, ABTS free radicals and DPPH free radicals. After the intervention of black garlic fermentation with *L. bulgaricus*, the clinical characteristics of GDM were improved by controlling weight gain of GDM patients, avoiding low birth weight babies and macrosomia, and improving birth quality of newborns. *L. bulgaricus* reduced the levels of FBG, 1hBG and 2hBG, and incidence of perinatal complications. *L. bulgaricus* reduced MDA level and increased the levels of SOD, GSH-PX and T-AOC levels. *L. bulgaricus* improves antioxidant capacity of black garlic in the prevention of GDM.

## Data availability

Full source data table available upon request from corresponding author via e-mail.

## Abbreviations

ABTS: 2,2′-azino-bis (3-ethylbenzenthiazoline-6-sulfonic) acid
BGP: black garlic polysaccharide
BMI: body-mass index
CFU: Colony forming units
DNS: dinitrosalicylic acid
DPPH: 2,2-diphenyl-1-picrylhydrazin
FBG: fasting blood glucose
FTIR: Fourier Transform infrared
GDM: gestational diabetes mellitus
GSH-PX: glutathione peroxidase
MDA: malondialdehyde
MRS: de-Man Rogosa Sharpe
NICU: neonatal intensive care unit
NMR: nuclear magnetic resonance
OGTT: oral glucose tolerance test
OH: hydroxyl radical
PCOS: polycystic ovary syndrome
SOD: superoxide dismutase
SR: scavenging rate
T-AOC: total antioxidant capacity
T2DM: type 2 diabetes mellitus
UV: ultraviolet
Vc: Vitamin C
1hBG: 1-h blood glucose
2hBG: 2-h blood glucose
